# *Bauhinia forficata* Link Protects HaCaT Keratinocytes from H_2_O_2_-Induced Oxidative Stress and Inflammation via Nrf2/PINK1 and NF-κB Signaling Pathways

**DOI:** 10.3390/plants14121751

**Published:** 2025-06-07

**Authors:** Qiwen Zheng, Xiangji Jin, Trang Thi Minh Nguyen, Jae-Woo Kim, Yong-Min Kim, Tae-Hoo Yi

**Affiliations:** 1 Graduate School of Biotechnology, Kyung Hee University, 1732 Deogyeong-daero, Giheung-gu, Yongin-si 17104, Gyeonggi-do, Republic of Korea; zhengqiwen@khu.ac.kr (Q.Z.); trangnguyen@khu.ac.kr (T.T.M.N.); 2 Department of Dermatology, Graduate School, Kyung Hee University, 26 Kyungheedae-ro, Dong-daemun, Seoul 02447, Republic of Korea; hyanghe112@khu.ac.kr; 3 Department of Convergent Biotechnology and Advanced Materials Engineering, Graduate School, Kyung Hee University, Yongin-si 17104, Gyeonggi-do, Republic of Korea; rlawodn0304@naver.com; 4 Department of Bio-Cosmetics, Semyung University, 65 Semyung-ro, Jecheon-si 27136, Chungcheongbuk-do, Republic of Korea; dragonroom@semyung.ac.kr

**Keywords:** *Bauhinia forficata* Link, antioxidant, anti-inflammatory, skin protection

## Abstract

Oxidative stress has been directly implicated in the pathogenesis of various skin disorders, making it a promising target for therapeutic intervention. *Bauhinia forficata* Link (BFL), commonly referred to as “plant insulin,” is well known for its antioxidant and antihyperglycemic properties; however, its potential role in skin protection remains unexplored. In this study, we investigated the protective effects of BFL against H_2_O_2_-induced oxidative stress and inflammation in HaCaT keratinocytes. The major phytochemical constituents of BFL were identified by high-performance liquid chromatography (HPLC). Its antioxidant capacity was evaluated using 2,2′-Azino-bis (3-ethylbenzothiazoline-6-sulfonic acid) (ABTS), 2,2-Diphenyl-1-picrylhydrazyl (DPPH), oxygen radical absorbance capacity (ORAC), and superoxide dismutase (SOD). In an H_2_O_2_-induced oxidative stress model, we assessed intracellular reactive oxygen species (ROS) levels and apoptosis using flow cytometry. Cellular respiration was analyzed using a Seahorse XFp analyzer, while molecular mechanisms were examined by reverse transcription polymerase chain reaction (RT-PCR) and western blotting. Our results demonstrated that BFL significantly reduced intracellular ROS levels and apoptosis, primarily by activating the nuclear factor erythroid 2–related factor 2 (Nrf2)/PINK1 pathway, which promoted mitochondrial quality control and redox homeostasis. Additionally, BFL suppressed inflammatory responses by downregulating the nuclear factor-κB (NF-κB) signaling pathway and reducing the secretion of pro-inflammatory cytokines interleukin (IL)-6, IL-1β, and tumor necrosis factor-alpha (TNF-α). These findings suggest that BFL is a potent antioxidant and anti-inflammatory agent, with potential as an adjunctive therapy for oxidative stress-related skin conditions.

## 1. Introduction

The skin, composed of the epidermis and dermis, serves as the body’s primary defense system against environmental insults and plays a vital role in maintaining internal homeostasis [1]. Among the various damaging stimuli, oxidative stress has been identified as a major contributor to skin pathophysiology [2]. Oxidative stress results from an imbalance between reactive oxygen species (ROS) production and antioxidant defenses, leading to molecular damage, mitochondrial dysfunction, and cellular dysregulation. These effects compromise skin barrier integrity, trigger pigmentary abnormalities, promote inflammation, and impair tissue repair mechanisms [3].

Growing evidence indicates that oxidative stress is directly involved in the pathogenesis of numerous skin disorders, including vitiligo [4], psoriasis [5], atopic dermatitis [6], acne vulgaris [7], and photoaging [8]. As such, targeting oxidative stress has emerged as a promising strategy for the prevention and treatment of skin-related diseases.

The detrimental effects of oxidative stress in the skin are largely mediated through the activation and amplification of inflammatory signaling pathways. Our previous study demonstrated that elevated ROS levels activate the MAPK/NF-κB signaling pathway, leading to excessive apoptosis in keratinocytes [8]. Once activated, NF-κB translocates into the nucleus and promotes the transcription of pro-inflammatory cytokines such as IL-1β, IL-6, and TNF-α, thereby establishing a pro-inflammatory microenvironment [9]. Sustained cytokine expression and immune cell infiltration may further perpetuate chronic inflammation and disrupt skin homeostasis [10].

One effective cellular mechanism to counteract oxidative stress is the activation of the Nrf2 pathway. Upon activation, Nrf2 translocates to the nucleus, where it binds to antioxidant response elements [11], inducing the expression of cytoprotective enzymes such as HO-1 and NQO-1 [12], which help neutralize excess ROS. Additionally, given that mitochondria are a primary source of intracellular ROS, restoring mitophagy via the Nrf2/PINK1 signaling axis plays a crucial role in maintaining redox balance [4]. This pathway facilitates the clearance of damaged mitochondria and supports mitochondrial function under stress conditions.

Due to their multi-target pharmacological profiles and high biocompatibility, plant extracts have gained increasing attention in dermatological research [13]. Bauhinia, a highly diverse genus comprising over 300 species, is widely distributed across tropical and subtropical regions [14]. Among them, *Bauhinia forficata* Link (BFL), a Fabaceae species native to South America, is an evergreen tree with morphologically distinctive bilobed leaves resembling hoof prints [15]. Traditionally, BFL has been extensively used in Brazilian folk medicine for the treatment of diabetes [16,17]. Phytochemical investigations have revealed that BFL is rich in a variety of secondary metabolites, including flavonoids, tannins, saponins, phenolic acids, and sterols [18]. Representative flavonoids such as hyperoside, kaempferol, and astragalin have been shown to exhibit potent antioxidant activity and demonstrate therapeutic efficacy in animal models of photoaging and atopic dermatitis [19,20,21]. Moreover, proteomic analysis has identified up to 131 proteins in BFL. The report further suggests that the pharmacological effects observed in diabetes-related studies are mainly attributed to its antioxidant properties rather than to a direct hypoglycemic effect [22]. These findings further indicate that the biological effects of plant extracts, especially their antioxidant capacity, arise from the synergistic action of multiple constituents rather than a single bioactive compound. Although the antioxidant potential of BFL has been well characterized, its protective effects against oxidative stress-induced skin damage have yet to be investigated.

Therefore, the present study aimed to evaluate the protective effects of BFL in H_2_O_2_-induced oxidative stress and inflammation in HaCaT keratinocytes, with a focus on its modulation of the Nrf2/PINK1 and NF-κB signaling pathways. Our findings suggest that BFL exerts significant cytoprotective effects and may serve as a promising adjunctive agent for managing oxidative stress-related skin disorders.

## 2. Results

### 2.1. Analysis of Chemical Contents of BFL

BFL exhibited high levels of total phenolic and flavonoid contents, quantified as 206.202 ± 0.189 mg gallic acid equivalent (GAE)/g extract and 202.474 ± 25.686 mg quercetin equivalent (QE)/g extract, respectively.

**Figure 1:**
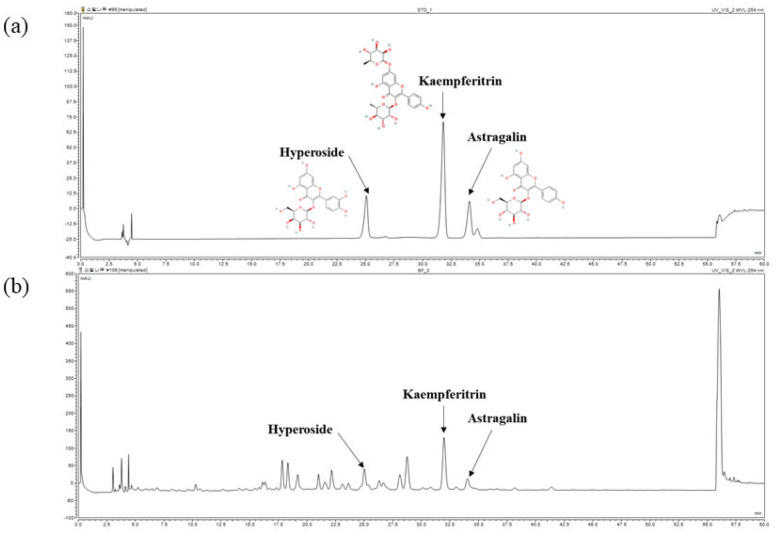
HPLC analysis of hyperoside, kaempferitrin, and astragalin (**a**); and the hyperoside, kaempferitrin, and astragalin content of BFL (**b**). The chromatogram was registered at 254 nm.

Furthermore, the chemical composition of BFL was analyzed using high-performance liquid chromatography (HPLC). As shown in Figure 1, hyperoside, kaempferitrin, and astragalin were identified as major components. The concentrations of hyperoside, kaempferitrin, and astragalin in BFL were determined to be 3.96 ± 0.04 mg/g, 25.86 ± 0.16 mg/g, and 2.85 ± 0.01 mg/g, respectively.

### 2.2. Antioxidative Activities of BFL

To comprehensively evaluate the antioxidant capacity of BFL, four distinct assays—DPPH, ABTS, ORAC, and SOD-like activity—were employed, each reflecting a different aspect of antioxidative potential.

BFL demonstrated considerable antioxidant potential across all four assays (Table 1). Specifically, it inhibited DPPH and ABTS free radicals in a dose-dependent manner, with IC_50_ values of 22.60 ± 3.28 μg/mL and 49.57 ± 5.82 μg/mL, respectively. The ORAC value reached 891.40 ± 87.27 μmol Trolox equivalent (TE)/g dry weight, indicating a substantial capacity for peroxyl radical quenching. Moreover, the SOD-like activity assay yielded an IC_50_ of 60.54 ± 5.42 μg/mL, reflecting the extract’s ability to neutralize superoxide radicals and enhance endogenous antioxidant defense mechanisms.

### 2.3. Effect of BFL on Intracellular ROS Production in H_2_O_2_-Induced HaCaT Keratinocytes

**Figure 2:**
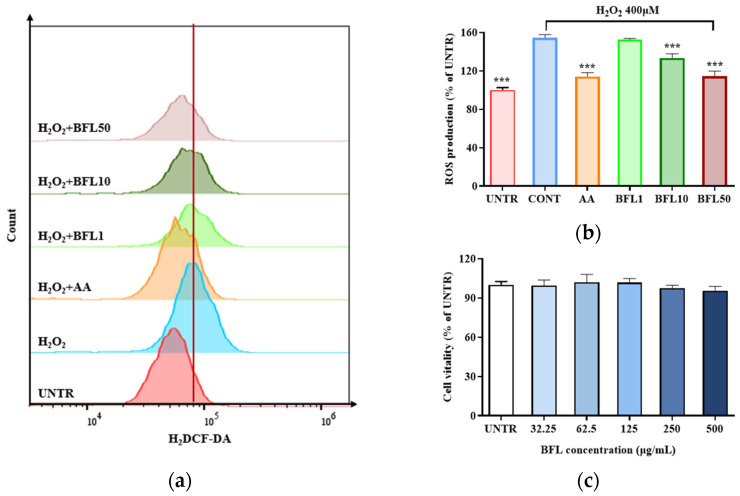
Intracellular ROS production in HaCaT keratinocytes treated with H_2_O_2_ and BFL, measured by flow cytometry (**a**); relative intensity histogram of ROS levels in H_2_O_2_-treated cells (**b**); and effect of BFL on cell viability (**c**). Results are reported as the mean ± SD from three independent experiments. AA (10 μM ascorbic acid) served as the positive control. BFL1, BFL10, and BFL50 denote *Bauhinia forficata* L. extract at 1, 10, and 50 μg/mL, respectively. *** *p*-value < 0.001 vs. CONT group.

As shown in Figure 2c, BFL treatment at concentrations ranging from 1 to 500 µg/mL had no significant effect on cell viability, maintaining viability above 90%. To achieve optimal efficacy with lower doses, subsequent experiments were conducted using BFL at 1, 10, and 50 µg/mL.

Figure 2a illustrates that ROS production increased rapidly by 54.51% in the H_2_O_2_-treated group compared to the untreated control. However, BFL treatment significantly reduced ROS levels relative to the H_2_O_2_-only group. Specifically, treatment with 50 µg/mL BFL resulted in a 25.82% reduction in ROS production. Similarly, 10 µM AA reduced ROS levels by 26.17%.

### 2.4. Effect of BFL on the Oxygen Consumption Rate (OCR) of H_2_O_2_-Induced HaCaT Keratinocytes

**Figure 3:**
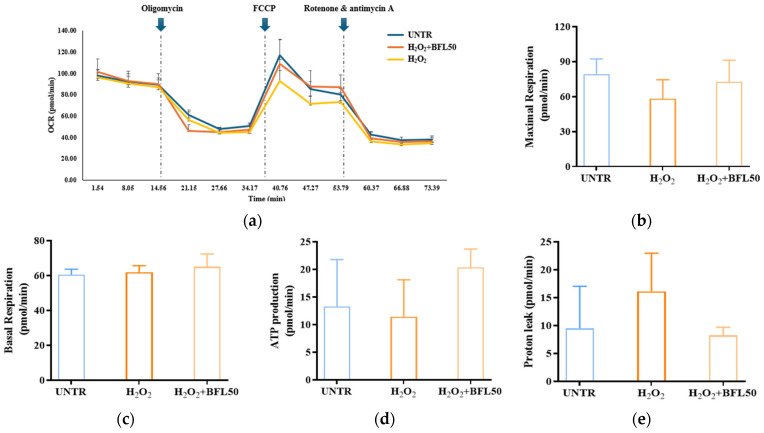
Oxygen consumption rate in HaCaT keratinocytes treated with H_2_O_2_ and BFL50, measured by Seahorse XFp Analyzer (**a**). Maximal respiration (**b**), basal respiration (**c**), ATP production (**d**), and proton leak (**e**). Results are reported as the mean ± SD from three independent experiments. BFL50 denote *Bauhinia forficata* L. extract at 50 μg/mL.

We evaluated the protective effect of BFL against H_2_O_2_-induced mitochondrial damage in HaCaT keratinocytes using OCR analysis (Figure 3a). As shown in Figure 3b,c, H_2_O_2_ treatment reduced maximal respiration by 26% while slightly increasing basal respiration by 2%. Concurrently, ATP production decreased by 13% (Figure 3d), and proton leak increased by 41% (Figure 3e), indicating mitochondrial dysfunction associated with oxidative stress. However, treatment with 50 μg/mL BFL significantly alleviated these impairments: maximal respiration was restored by 19%, ATP production increased by 77%, and proton leak decreased by 48%. Notably, basal respiration was further elevated by 4% compared to the H_2_O_2_-treated group.

These findings indicate that BFL treatment significantly reverses the H_2_O_2_-induced suppression of mitochondrial maximal respiration and ATP synthesis in HaCaT keratinocytes. By mitigating mitochondrial impairments caused by oxidative stress, BFL effectively prevents the ROS-mediated disruption of mitochondrial bioenergetics. Remarkably, BFL not only restores but further enhances basal respiration while reducing proton leakage, suggesting improved mitochondrial metabolic activity and respiratory efficiency. Collectively, these results demonstrate that BFL not only protects against oxidative stress-induced mitochondrial dysfunction but also promotes mitochondrial activation and cellular energy metabolism.

### 2.5. Effect of BFL on Nrf2/PINK1 Signaling Pathway in H_2_O_2_-Induced HaCaT Keratinocytes

**Figure 4:**
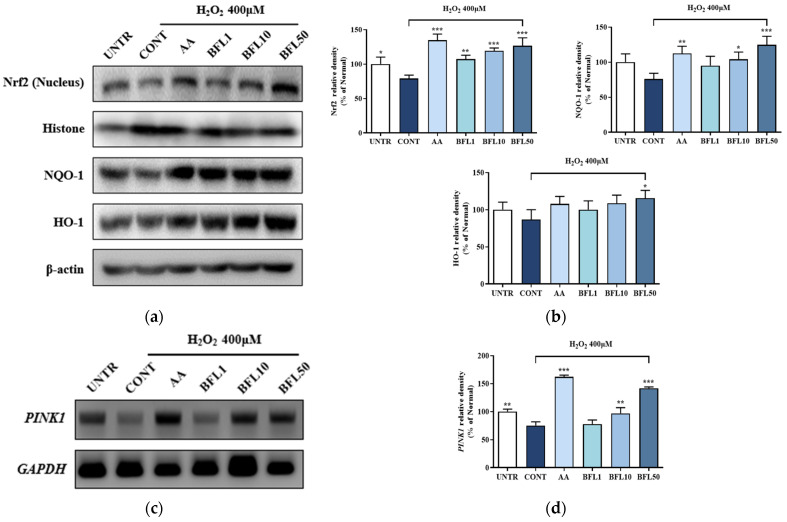
Protein expression of Nrf2, HO-1, and NQO-1 was assessed by Western blot (**a**). Band intensities were quantified by densitometry, normalized to the respective protein levels, and expressed as a percentage relative to the UNTR group (**b**). *PINK1* mRNA expression levels were analyzed by RT-PCR (**c**). Band intensities were quantified by densitometry, normalized to reference levels, and calculated as a percentage of the UNTR group (**d**). Results are reported as the mean ± SD from three independent experiments. AA (10 μM ascorbic acid) served as the positive control. BFL1, BFL10, and BFL50 denote *Bauhinia forficata* L. extract at 1, 10, and 50 μg/mL, respectively. * *p*-value < 0.05, ** *p*-value < 0.01, and *** *p*-value < 0.001 vs. CONT group.

To explore the underlying mechanism of BFL’s antioxidant effect, we investigated its impact on the Nrf2 signaling pathway. As shown in Figure 4a, H_2_O_2_ exposure reduced Nrf2 nuclear translocation by 20.8%, along with the downregulation of the downstream antioxidant enzymes HO-1 and NQO-1 by 13.18% and 23.91%, respectively (Figure 4b). Notably, treatment with 50 µg/mL BFL significantly enhanced Nrf2 nuclear translocation by 59.98% while also upregulating HO-1 and NQO-1 expression by 33.12% and 63.83%, respectively.

Furthermore, Zhao et al. reported that oxidative stress-induced mitochondrial dysfunction in keratinocytes could be alleviated via the Nrf2/PINK1 signaling axis [4]. In alignment with this, our study revealed that H_2_O_2_ reduced PINK1 mRNA expression by 24.86% (Figure 4d), while BFL treatment restored its expression in a dose-dependent manner. A concentration of 50 µg/mL BFL led to a 88.86% increase in PINK1 expression.

Collectively, these results suggest that BFL exerts its antioxidant effects not only through the activation of the Nrf2/HO-1/NQO-1 axis but also by restoring mitochondrial function via the Nrf2/PINK1 signaling pathway.

### 2.6. Anti-Apoptosis Effect of BFL on H_2_O_2_-Induced HaCaT Keratinocytes

Excessive apoptosis of HaCaT keratinocytes compromises skin structural integrity, weakens the epidermal barrier, accelerates skin aging, and may contribute to the development of skin disorders [8,23].

**Figure 5:**
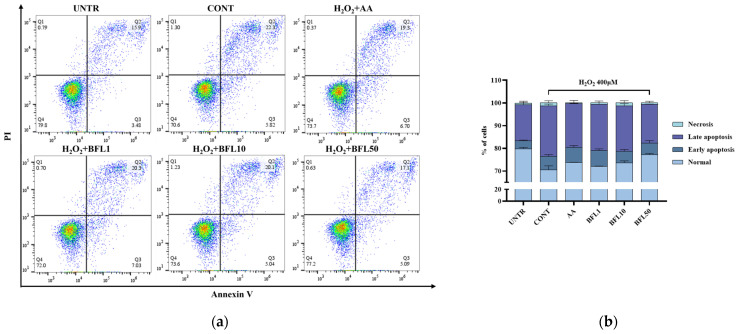
Apoptosis analysis in H_2_O_2_-treated HaCaT keratinocytes assessed by Annexin V-FITC/PI double staining, measured by flow cytometry (**a**); corresponding histograms illustrating apoptotic distribution (**b**). Results are reported as the mean ± SD from three independent experiments. AA (10 μM ascorbic acid) served as the positive control. BFL1, BFL10, and BFL50 denote *Bauhinia forficata* L. extract at 1, 10, and 50 μg/mL, respectively.

As shown in Figure 5a,b, the proportion of normal cells in the untreated group was 79.8%, with early apoptosis, late apoptosis, and necrotic cells accounting for 3.48%, 15.9%, and 0.79%, respectively. Following H_2_O_2_ treatment, the percentage of normal cells decreased to 70.6%, while early apoptosis, late apoptosis, and necrosis increased to 5.82%, 22.3%, and 1.3%, respectively. These results indicate that H_2_O_2_ induces substantial apoptosis in HaCaT keratinocytes.

Importantly, BFL treatment effectively alleviated this excessive apoptosis. After treatment with 50 µg/mL BFL, the proportion of normal cells recovered to 77.2%, while early apoptosis, late apoptosis, and necrosis decreased to 5.09%, 17.1%, and 0.63%, respectively.

### 2.7. Anti-Inflammation Effect of BFL on H_2_O_2_-Induced HaCaT Keratinocytes

**Figure 6:**
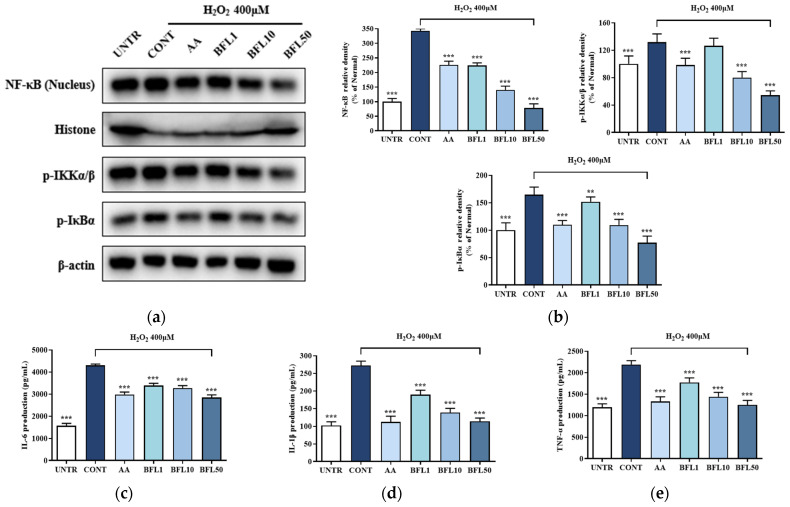
Protein expression levels of NF-κB, p-IKKα/β, and p-IκBα were assessed by Western blot (**a**). Band intensities were quantified by densitometry, normalized to reference levels, and calculated as a percentage of the UNTR group (**b**). Intracellular expression levels of IL-6 (**c**), IL-1β (**d**), and TNF-α (**e**) were measured by ELISA. Results are reported as the mean ± SD from three independent experiments. AA (10 μM ascorbic acid) served as the positive control. BFL1, BFL10, and BFL50 denote *Bauhinia forficata* L. extract at 1, 10, and 50 μg/mL, respectively. ** *p*-value < 0.01, and *** *p*-value < 0.001 vs. CONT group.

Following H_2_O_2_ stimulation, oxidative stress in HaCaT keratinocytes leads to phosphorylation of the IKK complex, resulting in the degradation of IκBα. This process enables NF-κB, particularly the p65 subunit, to translocate from the cytoplasm into the nucleus, thereby promoting the transcription of pro-inflammatory cytokines such as IL-6, IL-1β, and TNF-α. As shown in Figure 6a,b, exposure to H_2_O_2_ increased the phosphorylation of IKKα/β and IκBα by 31.83% and 64.66%, respectively, while NF-κB nuclear translocation rose by 242.71%.

Remarkably, BFL treatment significantly reversed these effects. Treatment with 50 µg/mL BFL reduced IKKα/β and IκBα phosphorylation by 58.74% and 64.66%, respectively, and decreased NF-κB nuclear translocation by 53.14%. These effects were notably stronger than those observed with the positive control group treated with AA.

Furthermore, ELISA analysis confirmed the anti-inflammatory potential of BFL (Figure 6c–e). H_2_O_2_ treatment markedly increased the secretion of IL-6, IL-1β, and TNF-α by 172.52%, 166.67%, and 83.51%, respectively. However, BFL effectively suppressed the secretion of these cytokines, with the 50 µg/mL dose reducing IL-6, IL-1β, and TNF-α levels by 33.85%, 140.15%, and 75.26%, respectively.

## 3. Discussion

With increasing environmental complexity, the skin is continuously exposed to a broad range of harmful exogenous stressors, including UV radiation (particularly UVA and UVB); air pollutants such as PM2.5, ozone, and nitrogen dioxide; microbial pathogens, including *Cutibacterium acnes* and *Staphylococcus aureus*; as well as tobacco smoke [7,8,23]. Despite their diverse nature, these environmental stressors commonly converge on a shared intracellular response cascade driven by the excessive accumulation of ROS [13]. Such oxidative perturbations compromise cutaneous homeostasis and increase susceptibility to various skin disorders. Growing evidence supports that the topical application of antioxidant-based skincare formulations can effectively neutralize ROS and mitigate environmentally induced skin damage [24,25]. Based on this, our study investigates the potential of BFL as an adjunctive therapeutic agent for the prevention and treatment of oxidative stress-related skin diseases.

In addition to their structural role, keratinocytes act as redox-sensitive immunomodulatory cells capable of mounting rapid responses to environmental challenges [26]. This dual functionality underscores their central role in the initiation and progression of oxidative stress-mediated skin pathology [27]. Accordingly, targeting the Nrf2 signaling pathway in keratinocytes has emerged as a promising strategy to attenuate oxidative injury in dermatological contexts [28]. Several phytochemicals have been shown to modulate this pathway effectively. For instance, syringic acid alleviates *C. acnes*-induced inflammation by activating Nrf2 and suppressing ROS accumulation [7], while gentiopicroside reduces ROS levels in TNF-α/IFN-γ-stimulated keratinocytes by regulating the Keap1–Nrf2 axis in an atopic dermatitis model [29]. Consistent with these findings, our data (Figure 2 and Figure 4) demonstrate that BFL activates Nrf2 signaling and significantly reduces intracellular ROS levels in H_2_O_2_-exposed keratinocytes.

Beyond cytosolic antioxidant defense, mitochondria play a pivotal role in redox regulation, serving as both the main source of ATP production and a primary endogenous generator of ROS during aerobic respiration [30]. However, mitochondria are particularly vulnerable to oxidative stress [31]. Elevated ROS levels can disrupt calcium homeostasis, trigger the release of pro-apoptotic factors, impair ATP synthesis, and ultimately lead to mitochondrial dysfunction [32]. In addition, defective clearance of oxidized mitochondrial DNA may result in persistent mitochondrial impairment and apoptosis. Thus, mitochondrial damage is a key pathological mechanism in oxidative stress-related skin disorders.

Previous studies have shown that the activation of Nrf2 enhances mitophagy, facilitating the selective removal of damaged mitochondria and restoration of mitochondrial quality [32]. Zhao et al. further demonstrated that the Nrf2/PINK1 axis is essential for regulating mitophagy and maintaining mitochondrial homeostasis in the epidermis [4]. In line with this, our results (Figure 3, Figure 4 and Figure 5) reveal that BFL enhances mitochondrial stability and function through the activation of the Nrf2/PINK1 pathway, thereby alleviating oxidative stress-induced keratinocyte apoptosis. These findings collectively support the role of BFL as a redox-regulating candidate for the prevention and management of skin disorders driven by oxidative stress.

While Nrf2 activation constitutes a key defense mechanism against oxidative damage, increasing evidence suggests that the effective suppression of inflammation also requires the modulation of pro-inflammatory signaling cascades, particularly the NF-κB pathway. Several plant-derived compounds have demonstrated the ability to alleviate oxidative stress-associated skin inflammation by targeting this pathway. For example, apigenin, a flavonoid found in Chamomilla recutita, was shown to inhibit UVB-induced NF-κB nuclear translocation and downregulate IL-6 and TNF-α in HaCaT keratinocytes, thereby attenuating photo-induced skin inflammation [33]. Likewise, resveratrol, a polyphenol abundant in grapes, suppressed NF-κB activation and cytokine release in PM-challenged keratinocytes by blocking IKK phosphorylation [34]. In models of atopic dermatitis, luteolin has also been reported to exert anti-inflammatory effects via the dual inhibition of MAPK and NF-κB signaling [35].

Building on this framework, our study revealed that BFL treatment significantly downregulated the NF-κB signaling cascade in H_2_O_2_-stimulated HaCaT keratinocytes. Specifically, we observed the reduced phosphorylation of IKKα/β and IκBα, as well as the suppressed nuclear translocation of NF-κB (Figure 6). These molecular changes correlated with a marked reduction in the expression of pro-inflammatory cytokines IL-6, IL-1β, and TNF-α. Taken together with our previous findings on Nrf2 activation, these results suggest that BFL exerts a coordinated dual-action effect by enhancing antioxidant defenses and concurrently suppressing inflammation, a profile consistent with other well-characterized phytochemicals.

## 4. Materials and Methods

### 4.1. Chemicals and Reagents

Fetal bovine serum (FBS), penicillin-streptomycin, and Dulbecco’s modified Eagle’s medium (DMEM) were bought at Gibco RBL (GrandIsland, NY, USA). Ascorbic acid, dexamethasone, 5-diphenyltetrazolium bromide (MTT), Folin-Ciocalteu reagent, gallic acid, and quercetin acid were bought from Sigma-Aldrich (St. Louis, MO, USA). Organic solvents were purchased from Daejung Chemical & Metal and Samchun Chemical (Siheung, Seoul, Republic of Korea). Sigma-Aldrich was used to acquire inorganic salts.

### 4.2. Sample Preparation

In total, 100 g of 40-mesh powdered green leaves of *Bauhinia forficata* L., sourced from Ecuador (Ecuadorian Rainforest, LLC, Clifton, NJ, USA), was extracted with 500 mL of 70% ethanol using a Twist shaker for 24 h at 24 °C. All extracts were combined and filtered twice through Whatman filter paper (Whatman, Maidstone, Kent, UK). The filtrate was then concentrated under reduced pressure at 40 °C using a rotary vacuum evaporator (Eyela World, Tokyo Rikakikai Co., Ltd., Tokyo, Japan). The final yield of BFL extract was 16.27%. A voucher specimen (No. 2021315013) was deposited at the Quality Standardization-Based Botanical Drug Development Center, Kyung Hee University, Yongin-si, Republic of Korea.

### 4.3. HPLC Analysis

BFL was prepared in 50% methanol at a concentration of 20 mg/mL. Standard compounds: 3-(β-D-Galactopyra-nosyloxy)-3′,4′,5,7-tetrahydroxyflavone (ChemFaces, Wuhan, Hubei, China), 3-(β-D-Glucopyranosyloxy)-4′,5,7-trihydroxyflavone (ChemFaces) (both in 30, 35, 40, 45, and 50 µg/mL), and 4′,5-Dihydroxy-3,7-bis(α-L-rhamnopyranosyloxy) flavone (ChemFaces) (150, 175, 200, 225, and 250 µg/mL) were prepared in methanol. HPLC was performed using a Dionex Chromeleon™ P580 system equipped with a UVD100 detector (Thermo Fisher Scientific™, Waltham, MA, USA). The column temperature was maintained at 32 °C, with a flow rate of 1.0 mL/min and an injection volume of 10 μL. To identify hyperoside, astragalin, and kaempferitrin, the mobile phase was A (0.1% formic acid in DW) and B (0.1% formic acid in acetonitrile). The gradient was linearly increased from 10% B to 20% B over 50 min. The detection wavelength was 254 nm. Linear correlations (R^2^) of the calibration curves for each compound were 0.999.

### 4.4. Antioxidant Analysis of BFL

The TPC and TFC of BFL were determined using standard colorimetric methods. For TPC, BFL (1 mg/mL) was reacted with Folin–Ciocalteu reagent and Na_2_CO_3_, and absorbance was measured at 625 nm. For TFC, BFL was sequentially reacted with NaNO_3_, AlCl_3_, NaOH, and distilled water, with absorbance recorded at 450 nm using a microplate reader (FilterMax F5, Molecular Devices, San Francisco, CA, USA). Gallic acid and quercetin were used to generate standard curves for TPC and TFC, respectively, both showing linearity (R^2^ = 1.000).

The antioxidant activity of BFL was evaluated using DPPH and ABTS radical scavenging assays. For DPPH, BFL at concentrations of 1–250 μg/mL was mixed with 0.2 mM DPPH solution in methanol and incubated for 30 min in the dark. Absorbance was measured at 517 nm. For ABTS, BFL samples (1–250 μg/mL) were reacted with ABTS solution diluted in ethanol for 15 min in the dark. Absorbance was recorded at 620 nm. Ascorbic acid was used as the positive control in both assays.

The ORAC was assessed using the OxiSelect™ ORAC Activity Assay Kit (Cell Biolabs, San Diego, CA, USA) according to the manufacturer’s protocol. Trolox was used as a reference standard antioxidant. Specifically, the method followed our previously established protocol [8].

SOD activity in BFL was measured using the EZ-SOD assay kit (DoGenBio, Seoul, Republic of Korea) following the manufacturer’s instructions.

### 4.5. Cell Culture and Treatment

HaCaT keratinocytes were supplied by Sciencell (Carlsbad, CA, USA). The cells were cultured in an incubator, at 37 °C and 5% CO_2_. Cell culture was performed with DMEM medium supplemented with 10% FBS and 1% penicillin-streptomycin.

To induce oxidative stress, HaCaT keratinocytes were cultured to approximately 80% confluence and pretreated with serum-free DMEM containing either 10 μM AA (positive control) or BFL at concentrations of 1, 10, or 50 μg/mL. After 1 h of incubation, 400 μM H_2_O_2_ was added directly to the medium and cells were co-incubated under the same conditions for the indicated experimental duration.

### 4.6. Cell Viability

After 24 h of sample treatment, the supernatant of each dish was removed, and 0.1 mg/mL MTT solution was added. After 4 h of incubation, the supernatant was aspirated, and DMSO was added to dissolve the formazan crystals. Absorbance was measured at 570 nm.

### 4.7. Measurement of the Cellular OCR

Cellular mitochondrial respiration was assessed using the Seahorse XF HS Mini Analyzer in conjunction with the Agilent Seahorse XF Cell Mito Stress Test Kit (Agilent Technologies, Santa Clara, CA, USA), following the manufacturer’s protocol and previously described methods [32]. Cells were seeded into Seahorse XF cell culture microplates and incubated overnight. Prior to analysis, the culture medium was replaced with assay-specific medium, and the plates were equilibrated in a non-CO_2_ incubator at 37 °C for 30 min. Mitochondrial stress testing was performed via the sequential injection of oligomycin (1.5 µM), FCCP (0.5 µM), and a combination of rotenone and antimycin A (0.5 µM each) through designated ports in the flux cartridge.

### 4.8. Flow Cytometry

Intracellular ROS levels were measured using 2′,7′-dichlorofluorescein diacetate (DCFH-DA; Sigma-Aldrich, St. Louis, MO, USA). After 24 h of treatment, cells were washed with PBS and incubated with 30 μM DCFH-DA for 30 min in the dark. Following staining, cells were collected and analyzed by flow cytometry (BD Accuri C6, BD Biosciences, Ann Arbor, MI, USA). Data were analyzed using FlowJo_v10.8.1 (FlowJo LLC, Ashland, OR, USA).

Cell apoptosis was assessed using the Annexin V-FITC/PI staining method. After 48 h of H_2_O_2_ exposure, both adherent and non-adherent cells were collected, centrifuged, and stained with Annexin V-FITC and propidium iodide (PI) according to the manufacturer’s instructions (Annexin V Apoptosis Detection Kit I; BD Biosciences). Fluorescence signals were detected via flow cytometry using FITC and PE channels.

### 4.9. Enzyme-Linked Immunosorbent Assay

The supernatants were collected after 72 h of sample treatment. The secretion of IL-1β, IL-6, and TNF-α was evaluated with commercially available ELISA kits (R&D Systems, Inc., Minneapolis, MN, USA), following the manufacturer’s instructions.

### 4.10. Reverse Transcriptase Polymerase Chain Reaction (RT-PCR)

After 24 h of sample treatment, the culture medium was removed, and cells were washed twice with 1× PBS. Total RNA was extracted using 700 μL of TRIzol reagent (Invitrogen, Carlsbad, CA, USA) per dish according to the manufacturer’s instructions. The concentration and purity of the RNA were quantified spectrophotometrically. Equal amounts of RNA (2 μg/μL) were reverse-transcribed using PCR premix with 0.5 μg/mL oligo(dT)_15_ and 0.5 μg/mL random hexamer primers (Bioneer Co., Daejeon, Republic of Korea). The cDNA synthesis was carried out at 70 °C for 5 min, followed by 42 °C for 60 min, and the reaction was terminated at 94 °C for 5 min. Amplified products were visualized by agarose gel electrophoresis and stained using a nucleic acid stain (NobleBio Inc., Gyeonggi, Republic of Korea) under UV illumination. GAPDH was used as the internal control gene.

### 4.11. Western Blotting

Following sample treatment, total cellular proteins were collected by washing cells with 1× PBS and lysing them in radioimmunoprecipitation assay (RIPA) buffer (Sigma-Aldrich, St. Louis, MO, USA) for 5 min. Nuclear and cytoplasmic proteins were separated using the NE-PER™ Nuclear and Cytoplasmic Extraction Reagents (Thermo Scientific, Waltham, IL, USA) according to the manufacturer’s instructions. Equal amounts of protein were separated on 10–15% SDS-PAGE and transferred onto nitrocellulose membranes (Amersham Pharmacia Biotech, Buckinghamshire, UK). Membranes were blocked with 3% BSA in TBST buffer (50 mM Tris-HCl, 150 mM NaCl, 0.1% Tween-20, and pH 7.4) for 30 min at room temperature, followed by overnight incubation at 4 °C with primary antibodies diluted in TBST. After 24 h, membranes were incubated with HRP-conjugated secondary antibodies for 1 h at 4 °C. Protein bands were visualized using Dyne ECL Pico Plus (DYNEBIO, Seongnam, Republic of Korea), and densitometric analysis was performed using ImageMaster™ 2D Elite v3.1 software (Amersham Pharmacia Biotech, Piscataway, NJ, USA).

### 4.12. Statistical Analysis

Results are reported as the mean ± SD from three independent experiments. The data were analyzed using a one-way ANOVA with Dunnett’s posttest, performed by GraphPad Prism 8 (GraphPad Software Inc., La Jolla, CA, USA). Statistical significance was set as follows: * *p*-value < 0.05, ** *p*-value < 0.01, and *** *p*-value < 0.001.

## 5. Conclusions

In this study, we demonstrated that BFL exerts potent protective effects against H_2_O_2_-induced oxidative stress and inflammation in HaCaT keratinocytes. BFL significantly attenuated intracellular ROS accumulation and mitochondrial dysfunction by activating the Nrf2/PINK1 signaling pathway, thereby enhancing antioxidant enzyme expression and supporting mitochondrial quality control. Additionally, BFL suppressed NF-κB activation and pro-inflammatory cytokine secretion, indicating its dual role as both an antioxidant and anti-inflammatory agent. These results suggest that BFL may serve as a promising adjunctive therapeutic agent for managing oxidative stress-related skin disorders. However, this study has several limitations. It was conducted solely in vitro using HaCaT keratinocytes, which may not fully represent in vivo skin physiology. Moreover, the pharmacokinetics, dermal permeability, and safety of BFL remain to be clarified. Further in vivo and clinical studies are necessary to validate its therapeutic potential.

## Figures and Tables

**Table 1:** Antioxidant properties of BFL.

	BFL	AA
**DPPH IC_50_ value (μg/mL)**	22.60 ± 3.28	5.58 ± 0.98
**ABTS IC_50_ value (μg/mL)**	49.57 ± 5.82	10.31 ± 1.08
**ORAC value (μmol TE/g DW)**	891.40 ± 87.27	1412.24 ± 172.38
**SOD-like IC50 value (μg/mL)**	60.54 ± 5.42	50.95 ± 3.25

Results are reported as the mean ± SD from three independent experiments.

## Data Availability

The data presented in this study are available in this paper.

## Abbreviations

BFL	*Bauhinia forficata* Link
DPPH	2,2-Diphenyl-1-picrylhydrazyl
ABTS	2,2′-Azino-bis (3-ethylbenzothiazoline-6-sulfonic acid)
TPC	Total Phenolic Content
TFC	Total Flavonoid Content
Nrf2	Nuclear Factor Erythroid 2–Related Factor 2
NF-κB	Nuclear factor-κB
IL	Interleukin
TNF-α	Tumor Necrosis Factor alpha
ROS	Reactive oxygen species
GAE	Gallic acid equivalent
QE	Quercetin equivalent
HPLC	High-performance liquid chromatography
SOD	Superoxide dismutase
TE	Trolox equivalent
ORAC	Oxygen radical absorbance capacity
OCR	Oxygen consumption rate
AA	Ascorbic acid
FBS	Fetal bovine serum
DMEM	Dulbecco’s modified Eagle’s medium
MTT	5-diphenyltetrazolium bromide
DCFH-DA	2′,7′-dichlorofluorescein diacetate
RT-PCR	Reverse transcriptase polymerase chain reaction
ELISA	Enzyme-linked immunosorbent assay
